# Comparison and correlation of commercial SARS-CoV-2 real-time-PCR assays, Ireland, June 2020

**DOI:** 10.2807/1560-7917.ES.2021.26.6.2002079

**Published:** 2021-02-11

**Authors:** Anne Carroll, Eleanor McNamara

**Affiliations:** 1 Public Health Laboratory-Health Service Executive-Dublin, Ireland

**Keywords:** SARS-CoV-2, RT-PCR, Cycle threshold

## Abstract

We report the performance of a variety of commercially available SARS-CoV-2 PCR kits, used in several different sites across Ireland to determine if C_t_ values across platforms are comparable. We also investigate whether a C_t_ value, a surrogate for calculated viral loads in the absence of viral culture of > 34 can be used to exclude SARS-CoV-2 infection and its complications. We found a variation in C_t _values from different assays for the same calculated viral load; this should be taken into consideration for result interpretation.

The interpretation of severe acute respiratory syndrome coronavirus 2 (SARS-CoV-2) real-time (RT)-PCR tests presents multiple conundrums with respect to viral load to cause infection and be infectious, age, clinical phase (presymptomatic, symptomatic, asymptomatic, resolution, re-infection or persistent positivity), testing purposes (diagnostic or surveillance), trending of previous test results if available and use of test result (e.g. for infection prevention control or occupational health purposes). In recent months, various publications have suggested that the use of cycle threshold (C_t_) values as surrogate for calculated viral load, may help in the management of patients [1-4].

In this study, we investigate if C_t_ values obtained by a variety of commercially available SARS-CoV-2 PCR kits, used in several different sites across Ireland, are comparable across platforms. We also explore whether a C_t_ value of > 34 [3], in the absence of viral culture, can be used to exclude SARS-CoV-2 infection.

## Ethical statement

No patient data or specimens were used in this study, therefore, ethical approval was not required.

## PCR platform comparison

In April 2020, Quality Control for Molecular Diagnostics (QCMD) produced a SARS-CoV-2 external quality assessment (EQA) panel [5]. The panel contained eight samples of which five were positive for SARS-CoV-2. Laboratories participating in the EQA were given the panel of samples without the respective information on positivity or negativity. Each laboratory processed the samples as if the provided material was viral transport media (VTM) from a SARS-CoV-2 inoculated swab, and tested the EQA panel according to their RT-PCR of choice and own laboratory procedures, then returned a ‘Detected’ or ‘Not detected’ result to QCMD. Following submission of results from all participants, in June 2020, QCMD provided a report to all participants, now detailing the digital (d)PCR log_10_ copies/mL of SARS-CoV-2 in the samples for reference purpose. The dPCR log_10_ copies/mL for the five SARS-CoV-2 positive samples were 4.3, 3.3, 4.3, 5.3, and 2.3 respectively.

In this study, we analyse the results of 16 participating clinical diagnostic laboratories across Ireland in more detail, using in particular the C_t_ values that they obtained with their RT-PCR assays for each of the five positive samples. For each laboratory, the C_t_ values for the five samples and the corresponding dPCR log_10_ copies/mL were employed to produce standard curves for each assay (or for each assay target). When several laboratories used a common assay, this allowed to assess the performance of the same assay across the platforms. Moreover, when laboratories used different assays, it was possible to compare outputs across assays. Six for 16 laboratories submitted data on more than one assay. In total nine assays with in total 15 gene targets were analysed (Table 1).

**Table 1:** Real-time PCR assays considered in the study, Ireland, June 2020 (n = 9 assays)

Assay	Number of laboratories	Number of gene targets	Genes targeted	Total datasets generated
GeneXpert (Cepheid, Sunnyvale, California, United States)	6	2	E gene N gene	12
Logix Smart (Co-Diagnostics, Inc, Salt Lake City, Utah, United States)	2	1	RdRp gene	2
Cobas 4800 (Roche Diagnostics, Basel, Switzerland)	2	2	ORF1a/b E gene	4
RealStar (Altona DiagnosticsGmbH, Hamburg, Germany)	1	1	E gene	1
genesig (Primerdesign, Southampton, Hants, United Kingdom)	4	1	ORF1a/b	4
RespiBio (Serosep, Limerick, Ireland)	2	1	RdRp gene	2
VIASURE (CerTest Biotec, Zaragoza, Spain)	3	2	ORF1a/b N gene	3 (2 genes combined)
Abbott Real*Time* SARS-CoV-2 (Abbott Park, Illinois, United States)	1	2	RdRp gene N gene	1 (2 genes combined)
Allplex SARS-CoV-2 (Seegene, Seoul, South Korea)	1	3	RdRp gene N gene E gene	3

E: envelope; N: nucleocapsid; ORF: open reading frame; RdRp: RNA-dependent RNA polymerase; SARS-CoV-2: severe acute respiratory syndrome coronavirus 2.

There were a number of different RNA extraction systems, either prior to the RT-PCR or incorporated within this procedure (no external extraction for GeneXpert (Cepheid)). Each of these systems used a different amount of sample (200 μL to 750 μL), with a varying portion of the total recovered RNA as subsequent PCR template. A standard curve was created in Microsoft Excel for each assay (one for each gene target). The standard curve was created by plotting the C_t_ value for each of the five samples against the dPCR log_10_ copies/mL provided by QCMD. R^2^ values of all the assays ranged from 0.9497 to 0.9997, with a mean of 0.9885. From each standard curve, estimated viral load (log_10_ copies per mL) was extrapolated (using the equation of the individual standard curve) for each C_t_ value. The Abbott Real*Time* SARS-CoV-2 assay reports cycle number (CN) values, which are not equivalent to C_t_ values and thus are not directly comparable [6] and was therefore excluded from further analysis.

Mean C_t_ values for all assays and standard deviations were calculated across a range of log_10_ copies per mL values (Table 2 and 3). Where only one laboratory tested an assay, absolute values were used for comparison. Correlation of C_t_ results between the same assays used across different sites was good for all assays (mean: 1.6; standard deviation: 0–5.1) (Figure, Table 2). Data from all assays correlated with the internationally recognised 3.3 cycle difference for every 1log_10_ copies/mL change in viral load. However, there was a wide variation in C_t_ values for different assays for the same viral loads, 6.5 cycle difference (31–37.5) at 3log_10_ down to 4 cycle difference (22–26) at 6log_10_. But the range difference in C_t_ values between assays was stable across all log values (Figure). These data demonstrate that reporting C_t_ values per se can be misleading and is non comparative between different assays, unless the C_t_ value is correlated with the calculated viral load for the particular assay used and also reported.

**Table 2:** Average and standard deviation of C_t_ values at calculated SARS-CoV-2 viral loads of 3log_10 _to 8log_10_ copies/mL, Ireland, June 2020 (n = 8 assays)^a^

Assay		Average C_t_ @ 3log_10_ (SD)	Average C_t_ @ 4log_10_ (SD)	Average C_t_ @ 5log_10_ (SD)	Average C_t_ @ 6log_10_ (SD)	Average C_t_ @ 7log_10_ (SD)	Average C_t_ @ 8log_10_ (SD)
GeneXpert	E	32.8 (1.32)	29 (0.57)	26 (0.63)	22 (0.72)	19 (0.79)	15 (0.89)
	N	35 (1.34)	31 (0.27)	27 (0.21)	23 (0.20)	20 (0.22)	16 (0.28)
Logix Smart	RdRp	37.5 (0.70)	33 (0.29)	30 (0.14)	26 (0.05)	23 (0.20)	19 (0.40)
Cobas 4800	ORF1a/b	33 (0.19)	29 (0.02)	26 (0.03)	23 (0.13)	20 (0.2)	17 (0.27)
	E gene	33.5 (0.7)	30 (0.05)	27 (0.03)	24 (0.10)	22 (0.15)	18 (0.25)
RealStar^b^	E	31 (NA)	28 (NA)	25 (NA)	22 (NA)	19 (NA)	16 (NA)
genesig	ORF1a/b	36 (1.63)	33 (0.43)	29 (0.72)	26 (0.97)	22 (1.31)	19 (1.59)
RespiBio	RdRp	37 (1.41)	33 (0.03)	30 (0.22)	26 (0.54)	23 (0.79)	19 (1.12)
VIASURE	ORF1a/b N (combined)	37 (3.78)	33 (0.96)	30 (0.87)	26 (0.99)	23 (1.21)	19 (1.6)
Allplex SARS-CoV-2^b^	E	32 (NA)	29 (NA)	27 (NA)	24 (NA)	22 (NA)	19 (NA)
	N	34 (NA)	32 (NA)	29 (NA)	26 (NA)	23 (NA)	21 (NA)
	RdRp	36 (NA)	32 (NA)	28 (NA)	24 (NA)	19 (NA)	16 (NA)

Ct: cycle threshold; E: envelope; NA: not applicable; N: nucleocapsid; ORF: open reading frame; RdRp: RNA-dependent RNA polymerase; SARS-CoV-2: severe acute respiratory syndrome coronavirus 2; SD: standard deviation.

^a^ While a total of nine SARS-CoV-2 real-time PCR assays were used in the study-participating laboratories, the Abbott Real*Time* SARS-CoV-2 assay reports cycle number values, which are not equivalent to C_t_ values and thus are not directly comparable. This assay was therefore excluded from the analysis.

^b^ Absolute values for Altona and Seegene targets as only data from one laboratory.

**Table 3:** Average SARS-CoV-2 viral loads of log_10_ copies/mL and standard deviation at selected C_t_ values, Ireland, June 2020 (n = 8 assays)^a^

C_t_	Average log_10_ (standard deviation) (copies/mL)											
	GeneXpert (Cepheid)		Logix Smart (Co-Diagnostics Inc)	Cobas 4800 (Roche)		RealStar (Altona Diagnostics)^b^	genesig (Primerdesign)	RespiBio (Serosep)	VIASURE (CerTest Biotec)	Allplex SARS-CoV-2 (Seegene)		
	E	N	RdPd	E	ORF1a/b	E	ORF1a/b	RdPd	ORF1a/b, N (combined)	E^b^	N^b^	RdRp^b^
30	3.84 (0.54)	4.23 (0.27)	4.99 (0.19)	3.63 (0.07)	4.10 (0.11)	3.50 (NA)	4.77 (0.56)	5.02 (0.14)	4.95 (0.88)	3.77 (NA)	4.64 (NA)	4.48 (NA)
31	3.55 (0.53)	3.97 (0.28)	4.70 (0.24)	3.30 (0.09)	3.76 (0.13)	3.19 (NA)	4.48 (0.49)	4.74 (0.05)	4.67 (0.92)	3.37 (NA)	4.27 (NA)	4.24 (NA)
32	3.26 (0.52)	3.70 (0.30)	4.42 (0.29)	2.97 (0.12)	3.41 (0.16)	2.87 (NA)	4.19 (0.43)	4.46 (0.03)	4.38 (0.96)	2.98 (NA)	3.90 (NA)	4.01 (NA)
33	2.97 (0.50)	3.43 (0.32)	4.14 (0.34)	2.64 (0.14)	3.07 (0.19)	2.55 (NA)	3.91 (0.38)	4.17 (0.11)	4.10 (1.02)	2.58 (NA)	3.54 (NA)	3.77 (NA)
34	2.69 (0.50)	3.17 (0.34)	3.86 (0.39)	2.31 (0.17)	2.72 (0.22)	2.24 (NA)	3.62 (0.35)	3.89 (0.19)	3.82 (1.09)	2.19 (NA)	3.17 (NA)	3.53 (NA)
35	2.40 (0.49)	2.90 (0.36)	3.58 (0.44)	1.98 (0.19)	2.38 (0.24)	1.92 (NA)	3.33 (0.34)	3.61 (0.27)	3.53 (1.17)	1.79 (NA)	2.81 (NA)	3.30 (NA)
36	2.11 (0.49)	2.63 (0.38)	3.30 (0.49)	1.65 (0.22)	2.04 (0.27)	1.61 (NA)	3.05 (0.35)	3.32 (0.36)	3.25 (1.26)	1.40 (NA)	2.44 (NA)	3.06 (NA)
37	1.83 (0.54)	2.37 (0.27)	3.02 (0.19)	1.32 (0.07)	1.69 (0.11)	1.29 (NA)	2.76 (0.56)	3.04 (0.14)	2.97 (0.88)	1.00 (NA)	2.08 (NA)	2.83 (NA)

Ct: cycle threshold; E: envelope; NA: not applicable; N: nucleocapsid; ORF: open reading frame; RdRp: RNA-dependent RNA polymerase; SARS-CoV-2: severe acute respiratory syndrome coronavirus 2.

^a^ While a total of nine SARS-CoV-2 real-time PCR assays were used in the study-participating laboratories, the Abbott Real*Time* SARS-CoV-2 assay reports cycle number values, which are not equivalent to C_t_ values and thus are not directly comparable. This assay was therefore excluded from the analysis.

^b^ Absolute values for Altona and Seegene targets as only data from one laboratory.

**Figure:**
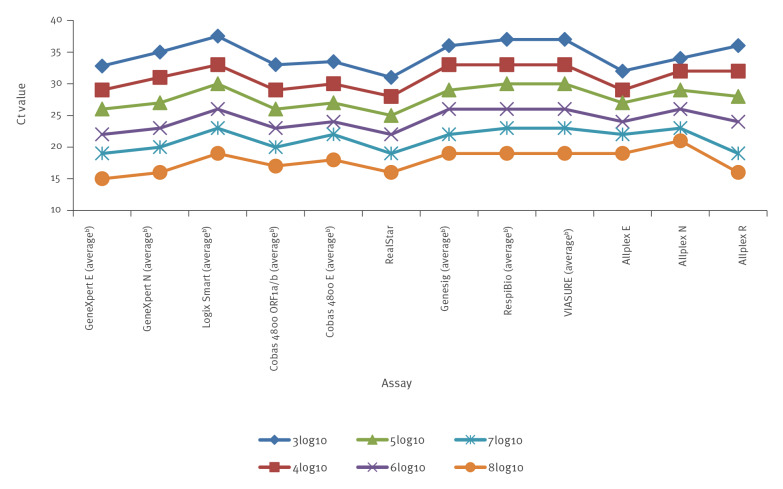
Average C_t_ values at SARS-CoV-2 viral loads of 3log_10_ to 8log_10_ copies/mL, Ireland, June 2020 (n = 8 assays)^a^

C_t_: cycle threshold; E: envelope; N: nucleocapsid; ORF: open reading frame; R: RNA-dependent RNA polymerase; SARS-CoV-2: severe acute respiratory syndrome coronavirus 2.

^a^ While a total of nine SARS-CoV-2 real-time PCR assays were used in the study-participating laboratories, the Abbott Real*Time* SARS-CoV-2 assay reports cycle number values, which are not equivalent to C_t_ values and thus are not directly comparable. This assay was therefore excluded from the analysis.

^b^ When more than one laboratory used an assay (or targets in the assay), the average C_t_s obtained across the laboratories are presented for each assay (or assay target).

Average C_t_ values at calculated viral loads of 3log_10_ to 8log_10_ copies/mL.

## Discussion

### Inferred viral loads

Tom et al. 2020 [3] noted that the issue of high C_t_ values can be problematic for clinicians, especially when there are less than 100 copies of the virus present, as this could reflect presymptomatic, early infection, late infection, persistent positivity or nonviable virus. From our data even at a C_t_ as high as 37, six of eight assays had at least one gene target correlating to calculated viral load of ≥ 100 copies/mL, although our study did not include viral culture, nor infer cultureability. The C_t_ cut off of 34 described by La Scola et al. [7] at which they propose patients can be discharged from isolation may need to be considered cautiously, as our data show that a C_t_ value of 34 has a range of calculated viral loads from 2.19log_10_ to 3.89log_10_ (equivalent to approximately 150 – > 7000 viral copies per mL (Table 3).

Our National coronavirus disease (COVID-19) guidelines [1,2] also describe the difficulty of interpreting positive ‘high’ (> 30) C_t_ PCR results from asymptomatic individuals, they too suggest that a C_t_ of 34 equates to < 100 copies/mL, however, our data indicate that the C_t_ value could be up to 38 for a calculated viral load of 100 copies/mL (data not shown).

A number of different RNA extraction systems were used by the participating laboratories, the effect this has on the results is unquantifiable. While it is a limitation of this study, our results support the view of Chik-Yan et al. [8] who state that differences in C_t_ values may be due to differences in specimen source or preparation or differences in cycling parameters and reagents, even though there is no significant difference in sensitivity. Indeed, for our data there was good correlation in C_t_ results using the same assay at different sites. Another limitation of the current study is that it investigated a small number of laboratories, with only one to six laboratories using the same assay (giving either absolute C_t_ values or averages based on small number of replicates). A larger study in the future would be useful to support these results.

Consensus on the correlation between C_t_ value and disease severity has not been reached, Sang Hyun Ra et al. [9] found that there was no significant difference in mean C_t_ values from symptomatic or asymptomatic cases, whereas Salvatore et al. [4] reported higher C_t_ values in asymptomatic individuals. Likewise Prubelli et al. [10] identified an increase in C_t_ values that correlated with a decrease in severe cases in Italy. While diagnostic test results play a role in identification and aid management of infected individuals, it is imperative to have a thorough understanding of the performance characteristics of individual PCR assays to aid the accurate interpretation of results [11].

### Cycle threshold values in patient management

Using C_t_ values to influence patient management is complex and must be done with caution. Including the C_t_ value on positive results may be confusing and misleading [12]. With no clearly defined infectious dose for SARS-CoV-2, viral culture not available routinely and C_t_ values differing by up to 6.5 cycles between platforms, one would question the value of the routine use of reporting C_t_ values for patient management. This is particularly challenging in a community testing setting, where frequently there is no accompanying patient clinical information to aid interpretation. However, this may be ameliorated in an acute hospital testing environment with access to patient clinical data. In this setting, analysis of C_t_ trends (either rising or decreasing) from repeat testing using the same assay may give more insight to an individual’s disease progression or resolution.

To aid the clinical interpretative value of COVID-19 PCR results, we agree with Tom et al. that binary reporting (‘Detected’ or ‘Not detected’) could be enhanced by the additional reporting of C_t_ value ranges in ‘high’, ‘medium’, ‘low’ categories . However, our data infer that such ranges if used, should be based on calculated viral loads of each assay used, not on absolute C_t_ values. The viral loads for ‘high’, ‘medium’ and ‘low’ categories would need to be defined. This reporting would be more accurate and informative to aid clinical and public health decisions, particularly when considered in the context of individual clinical data.
